# Cardiometabolic implications of triglyceride–glucose index, remnant cholesterol, and vitamin D in normoglycemic Arab adolescents: a cross-sectional study

**DOI:** 10.3389/fendo.2025.1674706

**Published:** 2026-01-07

**Authors:** Osama E. Amer, Shaun Sabico, Abdullah M. Alnaami, Kaiser Wani, Nasser M. Al-Daghri

**Affiliations:** Chair for Biomarkers of Chronic Diseases, Department of Biochemistry, College of Science, King Saud University, Riyadh, Saudi Arabia

**Keywords:** adolescents, cardiovascular diseases, insulin resistance, remnant cholesterol, triglyceride, vitamin D

## Abstract

**Introduction:**

The triglyceride-glucose (TyG) index, remnant cholesterol (RC), and vitamin D deficiency (VDD) are linked to cardiovascular diseases (CVDs) in adults. Nonetheless, their interplay in adolescents remains unclear. This observational study investigated associations of TyG index, RC, and vitamin D (VD) status with cardiometabolic risk factors in normoglycemic adolescents.

**Methods:**

In this cross-sectional, school-based study, 4,865 apparently healthy adolescents (12–17 years; 64.2% girls) were recruited from 60 randomly selected preparatory and secondary schools across Riyadh, Saudi Arabia (data collection: Sep–Nov 2023). Anthropometrics, blood pressure (BP), fasting glucose (FG) and lipid profile, and serum 25(OH)D were measured using standardized procedures. TyG and RC were calculated and analyzed as both continuous variables and by tertiles. Associations with cardiometabolic outcomes were assessed using multivariable linear and logistic regression adjusted for age and BMI; p-values were corrected for multiple testing using the False Discovery Rate (FDR) (Benjamini–Hochberg). Logistic models include Hosmer–Lemeshow and AUC metrics to assess calibration and discrimination.

**Results:**

After adjustment and FDR correction, higher TyG was robustly associated with adverse lipid profiles (lower HDL-C: β = -0.11, FDR-p < 0.001; higher RC: β = 0.40, FDR-p < 0.001) and with higher TC and LDL-C (FDR-p < 0.001). In tertile analyses, the highest TyG tertile was strongly associated with low HDL-C overall (OR T3 vs T1 = 1.8; FDR-p < 0.001) and was associated with vitamin D deficiency (VDD) and hypertension in boys (VDD in boys: OR = 1.6, 95% CI 1.2–2.2, FDR-p = 0.013; hypertension in boys: OR = 1.5, 95% CI 1.1–2.1, FDR-p = 0.010). Elevated RC (highest tertile) was independently associated with low HDL-C overall (OR T3 vs T1 = 0.7; FDR-p = 0.006) and with VDD in boys (OR = 1.5, 95% CI 1.1–2.0, FDR-p = 0.033). Logistic models showed acceptable calibration (Hosmer–Lemeshow p > 0.05) and modest discrimination (AUCs0.55-0.61, p < 0.001). Sex-stratified analyses revealed stronger TyG–25(OH)D inverse associations in boys than girls.

**Conclusion:**

TyG and RC are associated with VDD and cardiometabolic risks in normoglycemic adolescents, particularly in boys. Sexual dimorphisms were evident, with boys showing higher TyG index and VD levels, while girls had higher RC values. The TyG index was associated with hypertension and VDD in boys while linked to obesity and low HDL–C in girls. VD is inversely correlated with these risks, which warranting confirmation via randomized trials.

## 1 Introduction

Cardiovascular diseases (CVDs) remain the primary cause of morbidity and mortality among adults globally (1). Atherosclerosis, which initiates during childhood, is the primary pathological mechanism for CVDs (2). Hypercholesterolemia and dyslipidemia are of several childhood risk factors utilized for the prediction of CVDs in adulthood (3, 4). The triglyceride-glucose (TyG) index, calculated using fasting triglyceride (TG) and fasting glucose (FG) levels, has recently emerged as a reliable surrogate marker for insulin resistance (IR), itself an independent and critical contributor to CVDs (5, 6). Elevated TyG index values have been correlated with increased cardiovascular event rates, even after adjusting for conventional risk factors (6, 7).

VDD is a widespread public health concern, with global prevalence estimates ranging from 30% to 60% in both pediatric and adult populations (8). Its implications extend beyond bone health, as numerous studies link low VD levels with heightened risk for metabolic and cardiovascular conditions (9). VD also plays a regulatory role in lipid metabolism, influencing triglyceride concentrations and the broader lipid profile (10). These associations highlight the importance of addressing VDD as a potential contributor to cardiometabolic dysfunction.

The concept of residual CVD risk is complex, influenced by multiple factors such as remnant cholesterol (RC), lipoprotein(a), triglyceride-rich lipoproteins, and chronic inflammation (11). RC represents the cholesterol component within remnant lipoproteins, a subclass of triglyceride-rich lipoproteins. It is typically estimated by subtracting HDL–C and LDL–C from total cholesterol (TC) (12). RC is linked to various pathological processes, including inflammation, ischemic heart disease, and atherosclerosis (12). VDD has also been associated with elevated levels of RC (13). Although previously overlooked, RC has gained significant attention in recent years as a potential contributor to CVD risk factors.

Given the crucial roles of VD, TyG index, and RC in the regulation of glucose and lipid metabolism, it is logical to assess their interrelationship in the context of overall metabolic health. The majority of research has focused on the relationship between the TyG index, RC, and VD status in adults, with limited studies conducted on children and adolescents, particularly among Arab adolescents. Notably, cardiometabolic risk profiles differ substantially across ethnic groups due to variations in genetics, dietary habits, sun-exposure patterns, and cultural practices. However, data from Arab adolescents remain largely absent, representing a critical gap in the literature. Consequently, this study contributes uniquely to the field by evaluating TyG, RC, and VD status in one of the largest cohorts of Arab adolescents to date, providing population-specific insights that have not been reported previously. Given the distinct patterns of VD deficiency, lifestyle behaviors, and early cardiometabolic risk in the Middle East, examining these associations within this demographic is both timely and essential.

This study investigated the associations of TyG and RC with vitamin D status and cardiometabolic risk factors among normoglycemic Arab adolescents, thereby addressing a substantial regional evidence gap and expanding current pediatric cardiometabolic research beyond the Asian and Western contexts. Specifically, we sought to examine the relationships of TyG index and RC with VD status and other cardiometabolic risk factors, including obesity, dyslipidemia, and blood pressure, and explore sex-specific patterns in these associations. We hypothesized that higher TyG index and RC levels would be independently associated with a higher prevalence of VD deficiency and unfavorable cardiometabolic profiles.

## 2 Methods

### 2.1 Participants

This cross-sectional study included 4865 apparently healthy adolescents aged 12–17 years (64.2% girls), recruited from 60 randomly selected preparatory and secondary schools in Riyadh city, Saudi Arabia. Details of recruitment have been described elsewhere (14–17). Schools were selected using a multistage random sampling approach based on the official Ministry of Education registry for Riyadh. In the first stage, educational districts within the city were stratified geographically, and in the second stage, schools were randomly selected proportionally from boys’ and girls’ sectors within each district to ensure balanced representation. All selected schools were located within Riyadh, which is a fully urban metropolitan region; therefore, the study sample reflects the characteristics of urban school-attending adolescents in Saudi Arabia. Participants with prior diagnoses of diabetes, hypertension, CVD, or other chronic conditions, as well as those who had been prescribed medications to lower blood pressure or lipids, and those adhering to a particular dietary or weight management plan, as well as those who are taking VD supplementation were excluded. Ethical approval was obtained from the Institutional Review Board at the College of Medicine, King Saud University, Riyadh, Saudi Arabia (Nos. E–19–4239, E-21-6095). Written informed consent was obtained from parents or guardians, along with participant’s assent.

### 2.2 Anthropometric and biochemical analysis

All students were instructed to arrive in a fasted state for assessments held at their respective schools. Anthropometric measurements included height (cm), weight (kg), and body mass index (BMI, kg/m^2^). Blood pressure was recorded twice, 15 minutes apart, and the average of the two readings was taken. Pediatric-specific cutoff values were applied. Morning fasting blood samples were collected, and individuals with fasting glucose (FG) ≥100 mg/dL (5.6 mmol/L), indicating prediabetes or T2DM, were excluded in line with the American Diabetes Association criteria (18). This restriction was applied to ensure that the analyses focused exclusively on normoglycemic individuals, thereby minimizing potential confounding by overt glucose dysregulation and isolating the associations of TyG and RC within a metabolically healthy cohort. Standard laboratory analyses (Konelab 20 Thermo-Fischer, Espoo, Finland) were used to measure TC, HDL–C, TG, and FG levels (19). LDL–C was calculated using the Friedwald formula which has been validated for population studies but may introduce minor random error, particularly in samples with elevated triglycerides. Such measurement variability generally attenuates the magnitude of associations (biasing toward the null) rather than producing spurious correlations. Serum total 25(OH)D (VD) levels were measured using commercial electrochemiluminescence immunoassays (Roche Diagnostics, Germany) as previously done (20). All measurements were conducted by trained nursing and laboratory staff. The TyG index was calculated as Ln [fasting tri-glycerides (mg/dL) x fasting glucose (mg/dL)/2] (5). The RC was calculated as TC minus LDL–C minus HDL–C, [RC = TC - HDL–C - LDL–C] (12). No samples had triglycerides above 4.5 mmol/L.

### 2.3 Cut-offs and definitions

Lipid profile abnormalities were defined according to guidelines from the American College of Cardiology/American Heart Association (21); hypertriglyceridemia, serum TG levels ≥90 mg/dL (≥1.0 mmol/L); HDL–C levels <40 mg/dL (<1.0 mmol/L); and LDL–C levels ≥110 mg/dL (≥2.8 mmol/L). VD status was determined based on both national and regional guidelines (19), with deficiency defined as 25(OH)D levels <50 nmol/L (<20 ng/mL) and sufficiency as ≥50 nmol/L (≥20 ng/mL). Obesity classification was based on the International Obesity Task Force criteria (20). Although BMI does not directly differentiate between fat and lean mass or indicate fat distribution, it remains the most practical and validated screening tool for assessing obesity in large population-based adolescent studies. Hypertension was defined as SBP and/or DBP ≥95th percentile for age, sex, and height, in accordance with the 2017 guidelines from the American Academy of Pediatrics (22).

### 2.4 Data analysis

All statistical analyses were performed using SPSS software (version 22.0; IBM Corp., Chicago, IL, USA). Continuous variables were presented as means ± standard deviations (SDs) or medians (1st–3rd percentiles), depending on the distribution. Categorical data were summarized as frequencies and percentages. Distribution normality was tested using the Kolmogorov–Smirnov test, and variables showing non-normality were log-transformed.

TyG and RC values were categorized into tertiles for comparisons and regression analysis: TyG (T1: 6.87–8.04, T2: 8.05–8.44, T3: 8.45–9.44); RC (T1: 0.15–0.37, T2: 0.38–0.51, T3: 0.52–1.23). Group comparisons were conducted using independent T-tests, Analysis of Variance (ANOVA), or Kruskal–Wallis tests, where appropriate, for continuous variables and Chi-square test (χ^2^) for categorical variables.

Associations between the TyG index and continuous cardiometabolic parameters (SBP, DBP, HDL-C, LDL-C, TC, vitamin D, and RC) were examined using multiple linear regression models. Associations between TyG/RC tertiles and binary outcomes (obesity, hypertension, low HDL-C, and VDD) were evaluated using binary logistic regression models, reported as odds ratios (ORs) with 95% confidence intervals. Model calibration and discrimination were evaluated using the Hosmer–Lemeshow goodness-of-fit test (adequate fit: p > 0.05) and the area under the receiver operating characteristic curve (AUC, or C-statistic; acceptable discrimination: AUC ≥ 0.60). Odds ratios (ORs) with 95% confidence intervals (CIs) are presented for logistic models.

To address multiple testing, *Post-hoc* Bonferroni-adjusted p-values were applied where applicable, while Benjamini–Hochberg false discovery rate (FDR) correction was applied to the regression analyses p-values. A two-sided p < 0.05 was considered statistically significant unless otherwise noted. FDR correction was applied separately for each analytic family: one set of FDR-adjusted *p*-values was generated for the linear regression analyses, and a separate set was generated for the logistic regression analyses. This approach prevents inappropriate inflation of comparison counts across fundamentally different model types. For enhanced visualization of results, a forest plot was generated to summarize the adjusted odds ratios and 95% confidence intervals from all logistic regression models.

## 3 Results

A total of 4865 apparently healthy adolescents (12–17 years) were included in the analysis; 64.2% were girls. Anthropometric and biochemical characteristics are summarized in **Table 1**. Results revealed sexual dimorphisms where boys were slightly older and had significantly higher values for SBP, FG, TG, VD, TyG index, and RC. While girls had higher levels of TC, HDL–C, LDL–C, and DBP values. All differences remained significant after adjusting for age and BMI (adjusted p < 0.05).

**Table 1:** Descriptive characteristics of participants according to sex.

Parameter	All	Boys	Girls	Age and BMI adjusted p value
N (%)	4865	1742 (35.8)	3123 (64.2)	
Age (year)	14.48 ± 1.61	14.60 ± 1.55	14.41 ± 1.63	
BMI (kg/m^2^)	22.5 ± 5.7	22.8 ± 6.3	22.4 ± 5.5	
BMI Z-Score	-0.01 ± 0.98	0.04 ± 1.1	-0.02 ± 0.94	
SBP (mmHg)	115.4 ± 14.4	116.7 ± 14.4	115.3 ± 15.9	0.03
DBP (mmHg)	70.7 ± 10.8	68 ± 10.3	72.6 ± 12.2	<0.001
FG (mmol/l)	5.1 ± 0.7	5.2 ± 0.6	5.1 ± 0.6	<0.001
TG (mmol/l)	1.07 ± 0.4	1.14 ± 0.6	1.06 ± 0.4	<0.001
TC (mmol/l)	4.2 ± 0.8	4.1 ± 0.8	4.3 ± 0.9	<0.001
HDL–C (mmol/l)	1.02 ± 0.2	0.97 ± 0.2	1.04 ± 0.3	<0.001
LDL–C (mmol/l)	2.7 ± 0.7	2.6 ± 0.7	2.7 ± 0.8	<0.001
TyG index	8.3 ± 0.4	8.4 ± 0.5	8.3 ± 0.4	<0.001
RC (mmol/l)	0.52 ± 0.24	0.49 ± 0.21	0.47 ± 0.2	0.003
VD (nmol/l)	32.9 ± 14.5	39.4 ± 15.5	29.7 ± 14.5	<0.001
VDD N (%)	4106 (86.8)	1315 (78.2)	2791 (91.5)	<0.001

Group comparisons were conducted using independent-samples t-tests and χ^2^ tests for categorical variables; Bonferroni-adjusted p-values are reported. Data are presented as the means ± SDs, and the p value is significant at the 0.05 level. BMI, body mass index; SBP, systolic blood pressure; DBP, diastolic blood pressure; TG, triglycerides; TC, total cholesterol; HDL-C, high-density lipoprotein cholesterol; LDL-C, low-density lipoprotein cholesterol; FG, fasting glucose; TyG index, triglyceride-glucose index; RC, remnant cholesterol; VD, vitamin D.

**Figure 1:**
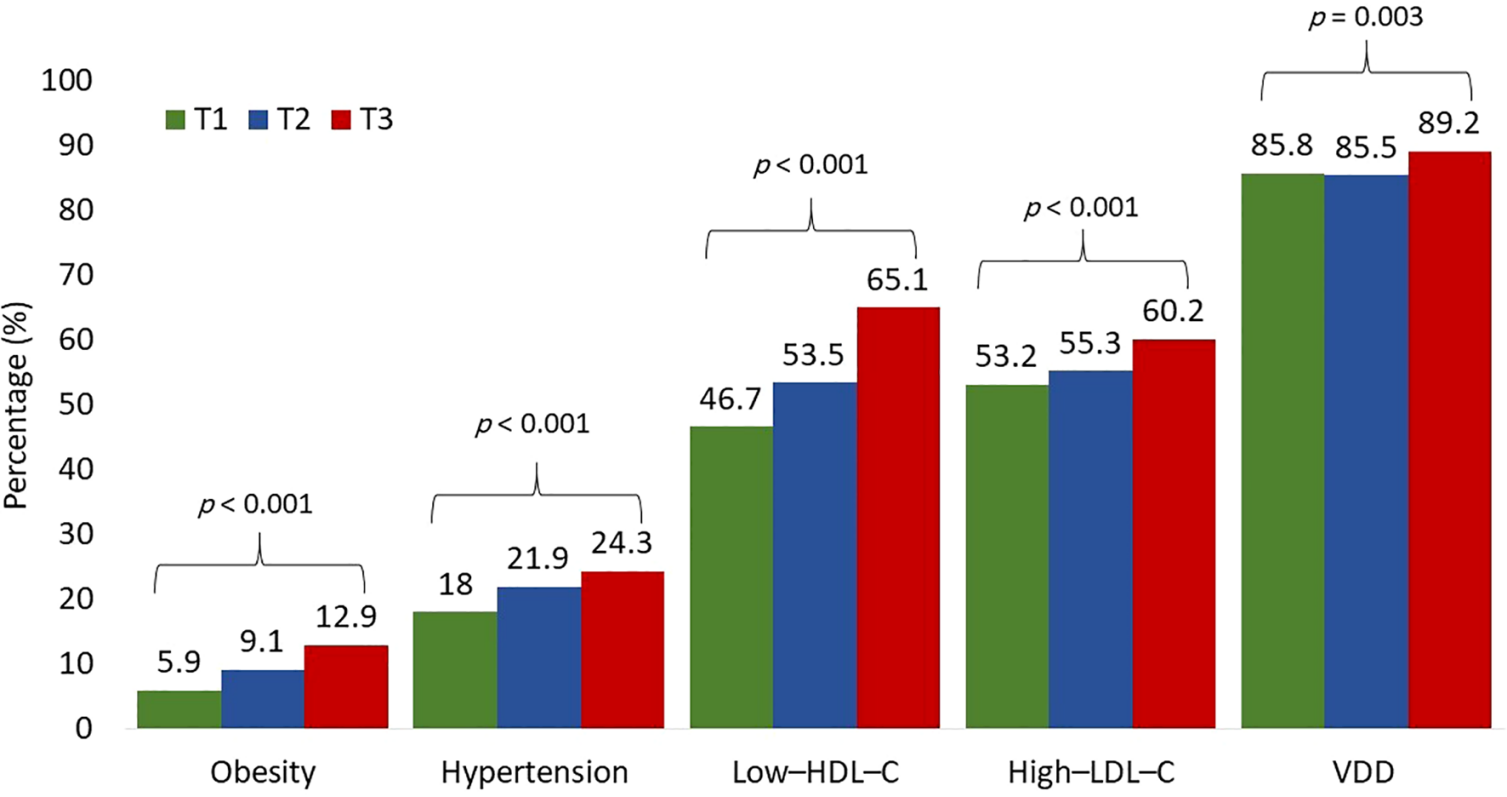
Prevalence of cardiometabolic risk factors according to the TyG index tertiles. Prevalence compared using χ^2^ tests.

**Figure 2:**
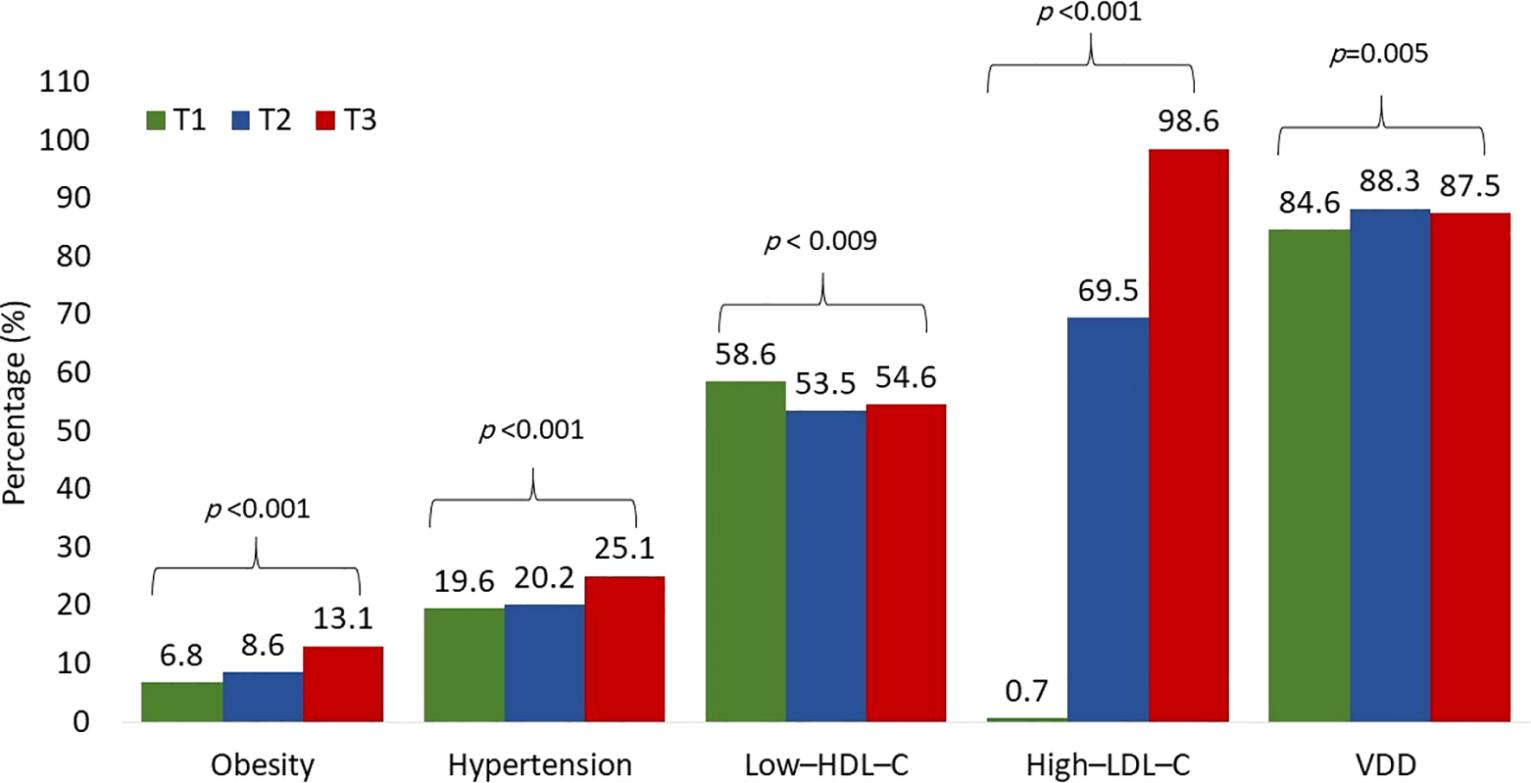
Prevalence of cardiometabolic risk factors according to the RC tertiles. Prevalence compared using χ^2^ tests.

**Table 2** shows the prevalence of cardiometabolic risk factors based on TyG index tertiles. the highest TyG index tertile had significant increase in BMI, SBP, TC, LDL–C, RC, and lower HDL–C levels were. As well as a significant increase in the prevalence of all measured cardiometabolic risk factors (obesity, hypertension, low–HDL–C, high–LDL–C, and VDD), **Figure 1**. Similarly, the highest RC tertile had significant increase in BMI, SBP, DBP, TyG index, TC, LDL–C, lower VD and HDL–C levels, and a significant increase in the prevalence of all measured cardiometabolic risk factors (**Table 3**; **Figure 2**). Notably, after adjustment for multiple comparisons, most of the associations remained significant, primarily those involving triglyceride-related indices and lipid parameters, as well as DBP and VD.

**Table 2:** Descriptive characteristics of participants according to TyG index tertiles.

Parameter	T1 (6.87-8.04)	T2 (8.05-8.44)	T3 (8.45-9.44)	Bonferroni age and BMI adjusted p value
N	1356	1862	1647	
Age (year)	14.60 ± 1.65	14.46 ± 1.59^a^	14.40 ± 1.58^a^	
BMI (kg/m2)	21.3 ± 5.1	22.3 ± 5.7^a^	23.9 ± 6.2^ab^	
BMI Z-Score	-0.22 ± 0.86	-0.05 ± 0.9^a^	0.23 ± 1.1^ab^	
SBP (mmHg)	114.4 ± 14.9	115.9 ± 15.6^a^	116.7 ± 15.6^a^	0.219
DBP (mmHg)	70.8 ± 12.1	71.3 ± 11.8	70.8 ± 11.4	0.046
FG (mmol/l)	4.8 ± 0.5	5.1 ± 0.5^a^	5.3 ± 0.6^ab^	<0.001
TG (mmol/l)	0.7 ± 0.1	0.9 ± 0.1^a^	1.6 ± 0.5^ab^	<0.001
TC (mmol/l)	3.9 ± 0.7	4.2 ± 0.7^a^	4.3 ± 1^ab^	<0.001
HDL–C (mmol/l)	1.1 ± 0.3	1.0 ± 0.3	0.96 ± 0.3^a^	<0.001
LDL–C (mmol/l)	2.5 ± 0.5	2.5 ± 0.7	2.7 ± 0.7^ab^	<0.001
TyG index	7.8 ± 0.2	8.2 ± 0.1^a^	8.8 ± 0.3^ab^	<0.001
RC (mmol/l)	0.31 ± 0.05	0.43 ± 0.07^a^	0.72 ± 0.22^ab^	<0.001
VD (nmol/l)	36.5 ± 15.9	32 ± 15.8	29 ± 14.9^ab^	0.007
VDD N (%)	1130 (85.8)	1553 (85.5)	1423 (89.2)^ab^	0.003

Group comparisons were conducted using Analysis of Variance (ANOVA) or Kruskal–Wallis tests where appropriate for continuous variables and χ^2^ tests for categorical variables, Bonferroni-adjusted p-values are reported. Data are presented as the means ± SDs, and the p value is significant at the 0.05 level. Superscripts ^a^ and ^b^ represented significance from T1 and T2 respectively. BMI, body mass index; SBP, systolic blood pressure; DBP, diastolic blood pressure; TG, triglycerides; TC, total cholesterol; HDL-C, high-density lipoprotein cholesterol; LDL-C, low-density lipoprotein cholesterol; FG, fasting glucose; TyG index, triglyceride-glucose index; RC, remnant cholesterol; VD, vitamin D; VDD, vitamin D deficiency.

**Table 3:** Descriptive characteristics of participants according to RC tertiles.

Parameter	T1 (0.15-0.37)	T2 (0.38-0.51)	T3 (0.52-1.23)	Bonferroni age and BMI adjusted p value
N	1608	1621	1636	
Age (year)	14.60 ± 1.60	14.46 ± 1.62^a^	14.38 ± 1.59^a^	
BMI (kg/m2)	21.8 ± 5.3	22.2 ± 5.4	23.6 ± 6.2^ab^	
BMI Z-Score	-0.14 ± 0.9	-0.07 ± 0.93	0.18 ± 1.08^ab^	
SBP (mmHg)	114.5 ± 13.9	114.9 ± 13.7	116.6 ± 15.4^ab^	0.31
DBP (mmHg)	69.8 ± 10.4	70.4 ± 10.6	71.9 ± 11.3^ab^	0.02
FG (mmol/l)	5.06 ± 0.31	5.1 ± 0.53	5.13 ± 0.54^ab^	<0.001
TG (mmol/l)	0.98 ± 0.4	1.03 ± 0.3^a^	1.2 ± 0.5^ab^	<0.001
TC (mmol/l)	3.4 ± 0.5	4.2 ± 0.3^a^	5.1 ± 0.5^ab^	<0.001
HDL–C (mmol/l)	1.03 ± 0.3	1.02 ± 0.2	1.01 ± 0.2^a^	<0.001
LDL–C (mmol/l)	1.94 ± 0.44	2.7 ± 0.24^a^	3.5 ± 0.44^ab^	<0.001
TyG index	8.20 ± 0.42	8.3 ± 0.34^a^	8.4 ± 0.41^ab^	<0.001
RC (mmol/l)	0.31 ± 0.05	0.4 ± 0.04^a^	0.72 ± 0.21^ab^	<0.001
VD (nmol/l)	33.5 ± 15	32.6 ± 14	32.4 ± 14^a^	0.03
VDD N (%)	1366 (85.6)	1325 (86.2)	1415 (88.7)^a^	0.02

Group comparisons were conducted using Analysis of Variance (ANOVA) or Kruskal–Wallis tests where appropriate for continuous variables and χ^2^ tests for categorical variables, Bonferroni-adjusted p-values are reported. Data are presented as the means ± SDs, and the p value is significant at the 0.05 level. Superscripts ^a^ and ^b^ represented significance from T1 and T2 respectively. BMI, body mass index; SBP, systolic blood pressure; DBP, diastolic blood pressure; TG, triglycerides; TC, total cholesterol; HDL-C, high-density lipoprotein cholesterol; LDL-C, low-density lipoprotein cholesterol; FG, fasting glucose; TyG index, triglyceride-glucose index; RC, remnant cholesterol; VD, vitamin D; VDD, vitamin D deficiency.

Vitamin D deficiency (VDD; 25[OH]D < 50 nmol/L) was highly prevalent in the cohort, affecting 86.8% of all participants. The prevalence differed significantly by sex, with girls exhibiting substantially higher rates of VDD compared with boys (91.5% vs. 78.2%, p < 0.001) (**Table 1**). VDD prevalence also increased progressively across TyG tertiles, from 85.8% in tertile 1 to 89.2% in tertile 3 (p = 0.003) (**Table 2**), and similarly across RC tertiles, from 85.6% in tertile 1 to 88.7% in tertile 3 (p = 0.02) (**Table 3**).

Results of linear regression models with the TyG index as a continuous predictor and continuous cardiometabolic outcomes (SBP, DBP, HDL–C, LDL–C, TC, VD, and RC) are provided in **Table 4** (overall and stratified by sex). After adjustment for age and BMI and application of FDR correction for multiple comparisons, in overall participants, higher TyG levels remained significantly positively associated with RC [β=0.4 (0.34, 0.44), FDR-p < 0.001], LDL–C, and TC, and significantly inversely associated with HDL–C [β = −0.11, 95%CI: -0.13, -0.09, FDR-p < 0.001]. Results showed differences in sex-stratified analyses, where association of TyG with LDL–C was more prominent in girls (FDR-*p* < 0.001), and an inverse association with VD was much stronger and significant in boys, where one-unit increment in TyG index corresponded to 3.13 nmol/l decrease in VD [β = −3.13, 95%CI: -4.8, -1.51), FDR-p = 0.0003].

**Table 4:** Linear Regression analysis for TyG with other parameters according to sex.

Parameter	Overall		Boys		Girls	
	β (95% CI)	FDR adj p	β (95% CI)	FDR adj p	β (95% CI)	FDR adj p
Model 1						
SBP	2.93 (1.93, 3.94)	<0.001	5.2 (3.8, 6.7)	<0.001	1.04 (-0.34, 2.42)	0.48
DBP	0.26 (-0.51, 1.03)	0.53	1.6 (0.6, 2.6)	0.004	0.04 (-1.01, 1.1)	0.94
HDL–C	-0.13 (-0.14, -0.11)	<0.001	-0.14 (-0.16, -0.11)	<0.001	-0.11 (-0.13, -0.08)	<0.001
LDL–C	-0.05 (-0.1, 0.002)	0.07	0.16 (.09, 0.24)	<0.001	-0.095 (-0.16, -0.03)	0.007
TC	0.30 (0.25, 0.36)	<0.001	0.54 (0.46, 0.61)	<0.001	0.17 (0.09, 0.25)	<0.001
VD	-1.9 (-2.94, -0.86)	<0.001	-4.5 (-6.1, -2.9)	<0.001	-1.5 (-2.8, -0.3)	0.02
RC	0.451 (0.45, 0.50)	<0.001	0.71 (0.6, 0.74)	<0.001	0.32 (0.24, 0.34)	<0.001
Model 2						
SBP	0.9 (-0.1, 1.9)	0.10	1.9 (0.5, 3.31)	0.0093	-0.41 (-1.79, 0.97)	0.57
DBP	-0.7 (-1.5, 0.1)	0.10	0.6 (-0.5, 1.6)	0.34	-0.76 (-1.84, 0.31)	0.18
HDL–C	-0.11 (-0.13, -0.09)	<0.001	-0.11 (-0.14, -0.09)	0.0000	-0.08 (-0.11, -.06)	<0.001
LDL–C	-0.09 (-0.15, -0.04)	<0.001	0.10 (0.03, 0.17)	0.0094	-0.12 (-0.19, -0.06)	<0.001
TC	0.27 (0.22, 0.33)	<0.001	0.49 (0.41, 0.57)	0.0000	0.16 (0.08, 0.24)	0.0001
VD	-1.4 (-2.5, -0.32)	0.01	-3.13 (-4.8, -1.51)	0.0003	-1.4 (-2.7, -0.12)	0.04
RC	0.4 (0.34, 0.44)	<0.001	0.61 (0.53, 0.71)	0.0000	0.31 (0.22, 0.41)	<0.001

Results derived from multiple linear regression models. FDR-adjusted p-values are shown. Model 1, no covariates were adjusted. Model 2 adjusted for age and BMI. P-value <0.05 considered significant. “FDR adj p” denotes False Discovery Rate–adjusted p-values using the Benjamini–Hochberg method. FDR-adjusted *p*-values were calculated within the family of linear regression tests only. SBP, systolic blood pressure; DBP, diastolic blood pressure; HDL-C, high-density lipoprotein cholesterol; LDL-C, low-density lipoprotein cholesterol; TC, total cholesterol; VD, vitamin D; RC, remnant cholesterol.

Multivariable logistic regression was used to determine odds ratios (OR) using TyG index and RC as independent variables and cardiometabolic risk factors as dependent variables, adjusted for age and BMI (**Tables 5**, **6**, respectively). Compared with the lowest tertile of the TyG index, age and BMI adjusted ORs showed that the highest TyG tertile was a significant risk factor for low HDL–C overall and in both sexes (FDR-p-value <0.001), VDD only in boys [OR = 1.6, 95%CI: 1.2–2.2, FDR-p = 0.013], and hypertension only in boys [OR = 1.5, 95%CI: 1.1–2.1, FDR-p = 0.01]. For the RC models (**Table 6**), highest RC tertile was associated with low HDL–C overall and only in boys after sex stratification [OR = 0.7, 95%CI: 0.5–0.9, FDR-p = 0.016] and with VDD only in boys [OR = 1.5, 95%CI: 1.1–2.0, FDR-p = 0.033]. Overall, models demonstrated acceptable calibration (non-significant Hosmer–Lemeshow tests) and modest discrimination (overall AUC values for TyG-index ranged between 0.55-0.70, p<0.001; AUC values for RC ranged between 0.54-0.66, p < 0.001).

**Table 5:** Cardiometabolic risk factors according to TyG index.

Risk factor	All			Boys			Girls		
	OR (95%CI), p value	FDR adj p	HL χ^2^ p value	OR (95%CI), p value	FDR adj p	HL χ^2^ p value	OR (95%CI), p value	FDR adj p	HL χ^2^ p value
Obesity									
1	1			1			1		
2	1.2 (0.9–1.7), *p* = 0.20	0.282	8.65,	0.8 (0.4–1.8), *p* = 0.6	0.757	9.03	1.3 (0.9–1.9), *p* = 0.2	0.257	2.85
3	1.4 (0.9–1.9), *p* = 0.05	0.121	*p* = 0.37	0.9 (0.5–1.9), *p* = 0.9	0.959	*p* = 0.34	1.4 (0.9–2.0), *p* = 0.08	0.151	*p* = 0.94
Hy*p*ertension									
1	1			1			1		
2	1.2 (0.9–1.4), *p* = 0.06	0.121	8.53,	1.1 (0.8–1.6), *p* = 0.5	0.638	5.11	1.2 (1.0–1.5), *p* = 0.1	0.19	6.89
3	1.2 (1.0–1.5), *p* = 0.03	0.09	*p* = 0.38	1.5 (1.1–2.1) *p* = 0.01	0.046	*p* = 0.746	1.0 (0.8–1.3), *p* = 0.9	0.959	*p* = 0.55
Low–HDL–C									
1	1			1			1		
2	1.3 (1.0–1.5), *p* < 0.01	0.009	8.12,	1.2 (0.9–1.6), *p* = 0.08	0.151	3.7	1.3 (1.0–1.5), *p* = 0.12	0.046	6.5
3	1.8 (1.6–2.2), *p* < 0.001	0	*p* = 0.42	2.4 (1.8–3.1), *p* < 0.001	0	*p* = 0.88	1.6 (1.3–2.0), *p* < 0.001	0	*p* = 0.59
VDD									
1	1			1			1		
2	0.9 (0.8–1.2), *p* = 0.60	0.959	15.07,	1.1 (0.8–1.5), *p* = 0.4	0.638	3.68	0.8 (0.6–1.1), *p* = 0.2	0.216	5.22
3	1.3 (1.0–1.6), *p* = 0.03	0.094	*p* = 0.06	1.6 (1.2–2.2), *p* = 0.003	0.013	*p* = 0.85	1.1 (0.8–1.6), *p* = 0.7	0.757	*p* = 0.73

Binary logistic regression models adjusted for age and BMI (obesity adjusted for age) was used to assess the cardiometabolic risk factors according to TyG index tertiles. Model calibration assessed using Hosmer–Lemeshow test and AUC. FDR-adjusted p-values are presented. Data *p*resented as OR (95% CI), *p*–value. FDR-adjusted *p*-values were calculated within the family of logistic regression tests (TyG models). “FDR adj p” denotes False Discovery Rate–adjusted p-values using the Benjamini–Hochberg method. OR, odds ratio; CI, confidence interval; AUC, area under the receiver operating characteristic curve; HL, Hosmer–Lemeshow goodness-of-fit test, these indicators assess model adequacy but are not intended to define diagnostic thresholds for TyG or RC.; HDL-C, high-density lipoprotein cholesterol; LDL-C, low-density lipoprotein cholesterol; VDD, vitamin D deficiency. Model 1 is unadjusted, and model 2 is adjusted for age and BMI.

**Table 6:** Cardiometabolic risk factors according to RC tertiles.

Risk factor	All			Boys			Girls		
	OR (95%CI), p value	FDR adj p	HL χ^2^ p value	OR (95%CI), p value	FDR adj p	HL χ^2^ p value	OR (95%CI), p value	FDR adj p	HL χ^2^ p value
Obesity									
1	1			1			1		
2	1.2 (0.9–1.7), *p* = 0.2	0.232	8.65	1.2 (0.7–2.5), *p* = 0.4	0.515	17.78	1.2 (0.8–1.7), *p* = 0.3	0.438	3.28
3	1.3 (0.9–1.8), *p* = 0.06	0.142	*p* = 0.40	1.3 (0.6–2.4), *p* = 0.4	0.506	*p* = 0.02	1.3 (0.9–1.8), *p* = 0.1	0.193	*p* = 0.92
Hy*p*ertension									
1	1			1			1		
2	1.01 (0.8–1.2), *p* = 0.8	0.914	16.23	0.8 (0.6–1.1), *p* = 0.1	0.203	9.22	1.2 (0.9–1.5), *p* = 0.1	0.221	10.97
3	1.2 (1.01–1.4), *p* = 0.04	0.108	*p* = 0.04	1.1 (0.8–1.4), *p* = 0.6	0.714	*p* = 0.32	1.3 (1.1–1.6), *p* = 0.02	0.063	*p* = 0.20
Low–HDL–C									
1	1			1			1		
2	0.8 (0.6–0.9), *p* = 0.002	0.016	5.6	0.8 (0.6–1.1), *p* = 0.2	0.203	6.88	0.8 (0.7–0.1), *p* = 0.03	0.097	7.5
3	0.7 (0.6–0.9), *p* < 0.001	0.006	*p* = 0.71	0.7 (0.5–0.9), *p* = 0.002	0.016	*p* = 0.55	0.9 (0.7–0.1), *p* = 0.09	0.193	*p* = 0.48
VDD									
1	1			1			1		
2	1.2 (0.9–1.5), *p* = 0.3	0.4	14.72	1.5 (1.1–1.9), *p* = 0.02	0.048	4.97	1.0 (0.7–1.4), *p* = 0.9	0.916	12.36
3	1.3 (1.06–1.6), *p* = 0.07	0.156	*p* = 0.07	1.5 (1.1–2.0), *p* = 0.006	0.033	*p* = 0.76	0.7 (0.5–1.0), *p* = 0.07	0.142	*p* = 0.14

Binary logistic regression models adjusted for age and BMI (obesity adjusted for age) was used to assess the cardiometabolic risk factors according to RC tertiles. Model calibration assessed using Hosmer–Lemeshow test and AUC. FDR-adjusted p-values are presented. Data *p*resented as OR (95% CI), *p*–value. FDR-adjusted *p*-values were calculated within the family of logistic regression tests (RC models). Data *p*resented as OR (95% CI), *p*–value. OR, odds ratio; CI, confidence interval; AUC, area under the receiver operating characteristic curve; HL, Hosmer–Lemeshow goodness-of-fit test, these indicators assess model adequacy but are not intended to define diagnostic thresholds for TyG or RC.; HDL-C, high-density lipoprotein cholesterol; LDL-C, low-density lipoprotein cholesterol; VDD, vitamin D deficiency. Model 1 is unadjusted, and model 2 is adjusted for age and BMI. P^*^: Bonferroni Corrected p value.

**Figure 3:**
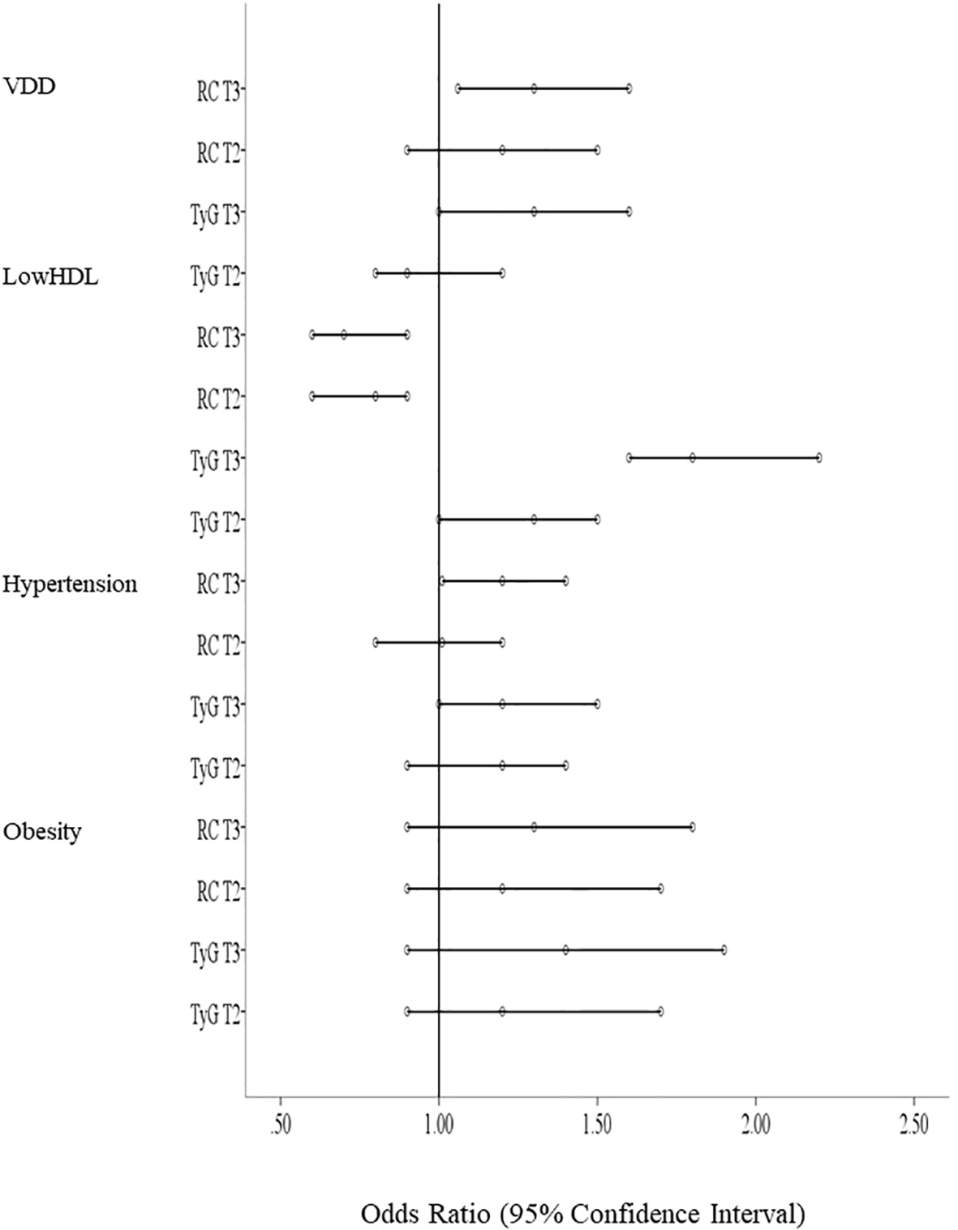
Associations of TyG and RC with cardiometabolic outcomes (forest plot). Forest plot showing odds ratios and 95% confidence intervals for associations between TyG and RC with cardiometabolic risk factors (obesity, hypertension, low–HDL–C, and VDD). See **Tables 5** and **6** for numeric odds ratios and confidence intervals.

To improve interpretability and minimize redundancy across multiple statistical outputs, the results from logistic regression models are additionally summarized in a consolidated forest plot (**Figure 3**). These regression models evaluate associations between TyG index, RC, and cardiometabolic risk factors. The inclusion of HL and AUC values serves to demonstrate model adequacy and reliability rather than predictive power.

In sex-stratified analyses (**Tables 4**–**6**), several associations showed modest differences in magnitude between boys and girls (for example, the inverse association between TyG and 25(OH)D was more pronounced in boys). These subgroup results are presented as descriptive findings because direct measures of sun exposure, clothing habits, and outdoor activity were not available to formally test mediation or effect modification.

## 4 Discussion

The present study investigated the associations between the TyG index, RC, and VD status in relation to cardiometabolic risk factors in a group of normoglycemic adolescents. The TyG index and RC were observed to be significantly associated with VDD and cardiometabolic risk factors among our study population. The findings indicate that higher TyG index values were significantly correlated with an increased prevalence of VDD, hypertension, low HDL–C, and high LDL–C. Additionally, there were significant parallel increases in several cardiometabolic risk factors, including RC, BMI, SBP, TG, TC, and LDL–C among participants with higher TyG index values. In contrast, both VD and HDL–C levels exhibited a significant decline with an increased TyG index. Furthermore, regression analyses revealed an inverse relationship between the TyG index and both VD and HDL–C, identifying the TyG index as a significant risk factor for VDD and low–HDL–C levels. The linear regression analyses further confirmed that TyG index, treated as a continuous variable, maintained robust associations with several cardiometabolic parameters even after stringent Bonferroni correction. These patterns were consistent across sex groups, highlighting the sensitivity of TyG index as a continuous indicator of dyslipidemia and metabolic changes in adolescents. These findings are consistent with earlier large-scale observational studies, such as one involving over 10,000 adolescents aged 10–19 years, which reported an inverse relationship between TyG and VD, independent of sex and T2DM status (23). Similarly, negative associations between TyG and VD have also been documented among middle-aged and elderly individuals with type 2 diabetes mellitus (T2DM) (24–26). Jia et al. additionally noted that this inverse relationship was significant only in male T2DM patients (25). Our findings reinforce the suggested role of VD in modulating insulin resistance and mitigating T2DM risk conditions closely tied to elevated cardiovascular morbidity and mortality due to concurrent risk factors such as hypertension, dyslipidemia, and obesity (27). VD contributes to insulin sensitivity by enhancing insulin receptor expression in liver, muscle, and adipose tissue (28). It may also exert epigenetic effects, regulating gene transcription relevant to insulin signaling, such as insulin receptor substrate (IRS), which has shown substantial upregulation in VD–treated high-fat diet mouse models (28). Furthermore, VD aids in insulin secretion by binding to its receptor on pancreatic β-cells and modulating calcium flux—both essential for glucose-induced insulin release (28, 29). Consequently, improving VD status may lower insulin resistance and serum insulin levels (28–30).

While the TyG index was inversely associated with VD and HDL–C levels, it was positively associated with TC, RC, LDL–C, SBP, DBP, and BMI. These findings align with previous research (12, 31). A recent large-scale meta-analysis evaluated randomized controlled trials (RCTs) assessed the effects of VD supplementation on LDL–C, HDL–C, TC, and TG, authors concluding that VD supplementation was negatively and significantly associated with LDL–C, TC, and TG, while simultaneously there were increments in HDL–C levels (32). Similarly, a meta-analysis focusing on RCTs indicated that the co-supplementation of VD and calcium in obese and overweight populations resulted in a substantial reduction in LDL–C, TG, and TC, alongside an elevation in HDL–C (33). Furthermore, another meta-analysis on RCT aimed to investigate the effects of VD supplementation on lipid profiles found a positive influence on VDD patients with hypercholesterolemia, who are at elevated cardiovascular risk (34). Additionally, previous studies have indicated that lower serum VD levels correlate with the metabolic syndrome and its components, particularly HDL–C concentrations in both men and women (35). Moreover, the Framingham Offspring Study revealed an increased risk of CVDs associated with VDD in hypertensive individuals, a relationship not observed in those without hypertension (36).

Similar to the TyG index in this study, RC was also significantly associated with hypertension, low–HDL–C levels, high–LDL–C levels, and VDD. In addition, the correlation between RC and TyG index was the highest compared to TyG and other measured cardiometabolic risk factors; a finding consistent with prior research indicating that TG, largely transported by remnant particles, increase proportionally with rising RC concentrations (37). Additionally, we observed that HDL–C levels decreased across RC tertiles, a trend attributed to cholesterol and TG exchange between remnant lipoproteins and HDL in the bloodstream (38).

Additionally, the present results indicate that RC is a risk factor for hypertension and low HDL–C levels. Recent studies have highlighted a strong association between RC and the prediction or diagnosis of hypertension, suggesting its potential utility as a biomarker for screening (39). Additionally, a recent large-scale study involving over 8 million Korean adults found that RC was valuable in predicting the future incidence of T2DM, independent of traditional lipid parameters, and authors reported that those in the highest quartile of RC exhibited a nearly two-fold risk of T2DM compared to those in the lowest quartile, notably, the increased risk for T2DM incidence was more pronounced among younger participants with elevated RC levels compared to older participants (40). Collectively, these findings highlight the significant relevance of RC levels to cardiometabolic disorders. Furthermore, a recent investigation revealed that a threshold concentration of RC levels 1 mmol/L (39 mg/dL) correlated with a two-fold increase in mortality attributed to cardiovascular causes (41). Moreover, another recent study indicated that elevated levels of both RC and LDL–C were linked to a greater risk of CVDs compared to the risk associated with either marker alone and increased RC concentrations, irrespective of LDL–C levels, were found to be associated with an increased risk of developing CVDs (42).

The physiological mechanisms underlying our observed associations may be explained through several interrelated pathways. VD was proposed to modulates glucose metabolism by enhancing insulin sensitivity, partially via upregulating insulin receptor expression and insulin signaling pathways (43) and by stabilizing intracellular calcium concentrations that are critical for insulin-mediated glucose uptake (44). It also exerts anti-inflammatory and immunomodulatory effects, which may indirectly be associated with improved metabolic homeostasis (35). RC is gaining prominence as a potent atherogenic marker due to its ability to penetrate the arterial intima, become trapped in the connective tissues, and be absorbed by macrophages without prior modification (45, 46). Its larger size also limits its capacity to diffuse back into circulation, enhancing its retention and pathogenicity. RC has been increasingly associated with atherosclerosis. Elevated non-fasting RC has been linked to ischemic heart disease in Mendelian randomization and observational analyses (47), and reviews highlight its atherogenic potential via direct lipid deposition, endothelial inflammation, and foam cell formation (48). Recent epidemiologic data also show that higher RC levels are associated with risk of coronary disease in women (49) Collectively, these mechanisms suggest that VD deficiency may impair insulin signaling and metabolic regulation, while elevated RC amplifies vascular inflammatory stress and lipid accumulation. Such interplay may underlie the inverse relationships between VD and both TyG index and RC observed in our adolescent cohort. These properties make RC an attractive clinical marker for early cardiometabolic risk detection. Prospective research should assess whether RC reduction can directly impact metabolic health and cardiovascular outcomes.

Sexual dimorphisms were observed in our study population, where boys exhibited higher values for the TyG index and VD levels, while girls showed elevated levels of RC. In addition, the TyG index was a risk factor for hypertension and VDD in boys but not in girls, whereas it was associated with obesity and low HDL–C levels in girls but not in boys. These findings align with the NHANES study, which indicated that elevated RC levels were more prevalent in girls than boys, while the incidence of low–HDL–C was greater in boys than in girls (50). Other studies have also noted higher levels of TC, LDL–C, and RC in females compared to males during adolescence (51, 52), likely influenced by estrogen’s regulatory effect on lipid metabolism (53, 54). This hormonal influence may partly explain the sex-specific differences in the association between TyG and VDD in our cohort. However, the observed sex differences, especially for VD, may reflect a combination of biological (e.g., pubertal hormonal effects) and behavioral/cultural influences (e.g., clothing coverage, time outdoors). Given that these behavioral measures were not directly recorded, the sex-stratified findings should be interpreted as hypothesis-generating and motivating for future, more granular investigations.

The high prevalence of VDD observed in this cohort aligns with previous findings from Saudi Arabia (55, 56). Consistent with regional and international literature, girls in our study demonstrated markedly higher deficiency rates than boys, a disparity attributed to differences in sun exposure, physical activity, and clothing patterns (57). Although the magnitude of variation across tertiles was relatively small, the directionality was consistent across two independent metabolic markers, supporting the concept that adolescents exhibiting greater early metabolic dysregulation may also be more likely to exhibit low VD levels. Similar clustering of low VD with dyslipidemia, low HDL-C, and insulin resistance has been reported in adolescent cohorts (58, 59), supporting the biological plausibility of these findings.

The present study was designed to assess associations rather than predictive thresholds. Although ROC analyses are valuable for defining clinically applicable cutoffs, our goal was to examine population-level relationships between TyG, RC, and cardiometabolic risk factors in a normoglycemic adolescent cohort. The observed AUC values confirm statistical associations but demonstrate limited discriminative ability, suggesting that these markers may serve as early correlates of metabolic risk rather than stand-alone diagnostic tools. Prospective studies with larger, diverse cohorts are warranted to establish clinically meaningful cutoff points for pediatric risk screening. As such, these markers should not be viewed as stand-alone screening tools in clinical practice. Instead, the present findings suggest that TyG and RC may serve as supportive indicators of adverse metabolic patterns, particularly in settings where more advanced assessments are not available. However, their clinical applicability for risk stratification remains limited without longitudinal validation. Any potential use of TyG or RC in clinical screening must therefore be considered preliminary, and prospective studies are required to determine their predictive value for incident hypertension, dyslipidemia, or VDD in adolescents.

Several limitations should be acknowledged. The cross-sectional design limits causal inference, and findings are primarily generalizable to urban, school-attending adolescents, with potential underestimation of associations due to the exclusion of individuals with impaired glycemia. BMI and indirectly calculated RC, while practical for large-scale studies, lack the precision of advanced adiposity and lipoprotein measurement techniques and may yield conservative effect estimates. Residual confounding cannot be excluded, as key variables including pubertal status and lifestyle factors were not assessed due to ethical and logistical constraints, despite statistical adjustments and Bonferroni correction. Nonetheless, this study is among the first to demonstrate associations between TyG, RC, and cardiometabolic risk in apparently healthy adolescents and represents the first such evidence in an Arab population.

## 5 Conclusions

Our study suggests that TyG index and RC as accessible and robust markers of cardiometabolic risk in adolescents, with TyG showing the strongest links, especially to VDD. The findings suggest VDD as a modifiable risk factor contributing to insulin resistance and dyslipidemia. Given their simplicity and cost-effectiveness, integrating TyG and RC into routine screening may facilitate early intervention. Furthermore, the early identification of at-risk adolescents using these indices could support timely preventive interventions including lifestyle changes and VD supplementation. These strategies could potentially mitigate the development of future cardiovascular diseases. A well-structured RCT to further investigate whether correcting VDD can positively influence TyG and RC levels, thereby reducing cardiometabolic risk. While the TyG index and RC demonstrated consistent associations with several cardiometabolic markers, their modest discriminatory ability underscores that these indices should currently be interpreted as correlational research tools rather than clinical screening tests. Further longitudinal and interventional studies are needed before establishing clinical thresholds or recommending their routine use in pediatric practice.

## Data Availability

The original contributions presented in the study are included in the article/**Supplementary Material**. Further inquiries can be directed to the corresponding author/s.
