# Supplementary material for: Cardiometabolic implications of triglyceride–glucose index, remnant cholesterol, and vitamin D in normoglycemic Arab adolescents: a cross-sectional study
Source: Front Endocrinol (Lausanne). 2026 Jan 7;16:1674706. doi: 10.3389/fendo.2025.1674706 (PMC12819213; doi:10.3389/fendo.2025.1674706)
Supplement: Supplementary file 1 [file Table1.docx]

S. table 1. Correlations of TyG index with other Parameters

| **Parameter** | **TyG** | | | **RC** | | |
| --- | --- | --- | --- | --- | --- | --- |
|  | **Overall** | **Boys** | **Girls** | **Overall** | **Boys** | **Girls** |
| Age | -0.05** | 0.05* | -0.11** | -0.06** | -0.09** | -0.04* |
| BMI | 0.21** | 0.33** | 0.12** | 0.14** | 0.24** | 0.09** |
| SBP | 0.01** | 0.17** |  | 0.05** | 0.05* | 0.05** |
| DBP |  | 0.07** |  | 0.08** |  | 0.09** |
| HDL–C | -0.21** | -0.26** | -0.15** | -0.03 |  | -0.05** |
| TC | 0.16** | 0.30** | 0.08** | 0.95** | 0.95** | 0.94** |
| LDL–C |  | 0.10** | -0.08** | 0.96** | 0.95** | 0.97** |
| RC | 0.23** | 0.40** | 0.16** |  |  |  |
| VD | -0.05** | -0.13** | -0.04* | -0.02 | -0.09** | 0.06** |

Note: Data presented as coefficient (R). * and ** represented p-value significant at 0.05 and 0.01 level respectively. BMI, body mass index; SBP, systolic blood pressure; DBP, diastolic blood pressure; HDL-C, high-density lipoprotein cholesterol; TC, total cholesterol; LDL-C, low-density lipoprotein cholesterol; VD, vitamin D.

S. table 2. TyG index, RC, and VD levels according to presence of CVDs Risk Factors.

| **Risk factor** | **VD** | **P-value** | **TyG** | **P-value** | **RC** | **P-value** |
| --- | --- | --- | --- | --- | --- | --- |
| **Obesity**  No  Yes | 34.5±16.4  32.3±14.7 | *P*<0.001 | 8.1±0.2  8.5±0.4 | *P*<0.001 | 3.1±0.7  3.3±0.8 | *P*<0.001 |
| **Hypertension** No  Yes | 34.1±16.2  32.7±14.8 | *P*=0.009 | 8.2±0.1  8.4±0.3 | *P*<0.001 | 3.1±0.6  3.3±0.8 | *P*<0.001 |
| **Low-HDL-C**  No  Yes | 34.4±16.1  33.2±15.8 | 0.01 | 8.2±0.4  8.4±0.5 | *P*<0.001 | 3.2±0.6  3.2±0.7 | P=0.2 |
| **High -LDL-C**  No  Yes | 33.8±16.1  33.7±15.8 | 0.6 | 8.3±0.5  8.3±0.4 | 0.8 | 2.5±0.4  3.7±0.5 | *P*<0.001 |
| **VDD**  No  Yes | 61.3±7.7  28.6±9.6 | *P*<0.001 | 8.1±0.3  8.3±0.5 | *P*<0.001 | 3.1±0.7  3.2±0.8 | 0.053 |

Note: Data are presented as the means ± SDs, and the p value is significant at the 0.05 level. HDL-C, high-density lipoprotein cholesterol; LDL-C, low-density lipoprotein cholesterol; VDD, vitamin D deficiency.

S. table 3. Cardiometabolic risk factors according to TyG as a continuous variable.

| **Parameters** | **Overall** | | **Boys** | | | **Girls** | | |
| --- | --- | --- | --- | --- | --- | --- | --- | --- |
|  | **Odd ratio (95% CI)** | **P–value** | **Odd ratio (95% CI)** | **P–value** | **Odd ratio (95% CI)** | | **P–value** |  |
| **Model 1** | | | | | | | | |
| Hypertension | 1.5 (1.3–1.8) | <0.001 | 1.9 (1.5–2.4) | <0.001 | 1.3 (1.1–1.6) | | 0.01 |  |
| Obesity | 2.1 (1.7–2.6) | <0.001 | 2.1 (1.5–3.1) | <0.001 | 2.4 (1.8–3.1) | | <0.001 |  |
| Low HDL–C | 2.3 (2.1–2.8) | <0.001 | 3.2 (2.5–4.1) | <0.001 | 2.1 (1.7–2.5) | | <0.001 |  |
| High LDL–C | 0.9 (0.8–1.1) | 0.9 | 1.3 (1.1–1.6) | 0.009 | 0.8 (0.7–1.1) | | 0.07 |  |
| VDD | 1.5 (1.2–1.8) | <0.001 | 1.9 (1.4–2.5) | <0.001 | 1.3 (0.9–1.8) | | 0.1 |  |
| **Model 2** | | | | | | | | |
| Hypertension | 1.2 (1.1–1.5) | 0.01 | 1.4 (1.1–1.8) | 0.01 | 1.1 (0.9–1.4) | | 0.4 |  |
| Obesity | 1 (0.8–1.3) | 0.9 | 0.8 (0.5–1.4) | 0.5 | 1.4 (1–1.9) | | 0.04 |  |
| Low HDL–C | 2.3 (1.9–2.6) | <0.001 | 2.7 (2.1–3.5) | <0.001 | 1.9 (1.6–2.3) | | <0.001 |  |
| High LDL–C | 0.9 (0.8–1) | 0.1 | 1.1 (0.9–1.4) | 0.3 | 0.8 (0.7–0.9) | | 0.04 |  |
| VDD | 1.3 (1.1–1.7) | 0.006 | 1.6 (1.2–2.1) | 0.001 | 1.3 (0.9–1.8) | | 0.2 |  |

Note: Data represent the OR (odds ratio) (95% CI), and the p value is significant at the 0.05 level. HDL-C, high-density lipoprotein cholesterol; LDL-C, low-density lipoprotein cholesterol; VDD, vitamin D deficiency. Model 1 is unadjusted, and model 2 adjusted for age and BMI.

S. table 4. General characteristics of participants according to BMI status.

| **Parameter** | **Normal BMI** | **Overweight/Obese** | **P value** |
| --- | --- | --- | --- |
| N (%) | 3114 (64) | 1751 (36) |  |
| Age (year) | 14.5±1.6 | 14.5±1.5 | 0.08 |
| SBP (mmHg) | 113±14 | 120±14 | <0.001 |
| DBP (mmHg) | 70±10 | 72±11 | <0.001 |
| FG (mmol/l) | 5.07±0.6 | 5.14±0.6 | <0.001 |
| TG (mmol/l) | 1.01±0.4 | 1.20±0.5 | <0.001 |
| TC (mmol/l) | 4.20±0.8 | 4.30±0.8 | <0.001 |
| HDL–C (mmol/l) | 1.05±0.3 | 0.96±0.2 | <0.001 |
| LDL–C (mmol/l) | 2.7±0.7 | 2.8±0.7 | <0.001 |
| TyG index | 8.24±0.4 | 8.42±0.4 | <0.001 |
| RC (mmol/l) | 0.45±0.2 | 0.54±0.2 | <0.001 |
| VD (nmol/l) | 33.5±14.8 | 31.9±13.8 | <0.001 |

Note: Data are presented as the means ± SDs, and the p value is significant at the 0.05 level. BMI, body mass index; SBP, systolic blood pressure; DBP, diastolic blood pressure; TG, triglycerides; TC, total cholesterol; HDL-C, high-density lipoprotein cholesterol; LDL-C, low-density lipoprotein cholesterol; FG, fasting glucose; TyG index, triglyceride-glucose index; RC, remnant cholesterol; VD, vitamin D.

S. table 5. General characteristics of participants according to VD status.

| **Parameter** | **Sufficient** | **Deficient** | **P value** | **BMI Adjusted p value** |
| --- | --- | --- | --- | --- |
| N (%) | 642 (13.2) | 4223 (86.8) |  |  |
| Age (year) | 14.4±1.6 | 14.5±1.5 | 0.1 |  |
| BMI (kg/m^2^) | 21.5±5.4 | 22.6±5.7 | <0.001 |  |
| BMI Z-Score | -0.18±0.94 | 0.02±0.99 | <0.001 |  |
| SBP (mmHg) | 112±13 | 116±14 | <0.001 | <0.001 |
| DBP (mmHg) | 69±10 | 71±11 | <0.001 | <0.001 |
| FG (mmol/l) | 5.03±0.6 | 5.09±0.6 | 0.005 | 0.01 |
| TG (mmol/l) | 1.03±0.4 | 1.08±0.4 | 0.004 | 0.07 |
| TC (mmol/l) | 4.20±0.8 | 4.22±0.8 | 0.3 | 0.5 |
| HDL–C (mmol/l) | 1.04±0.3 | 1.02±0.3 | 0.04 | 0.2 |
| LDL–C (mmol/l) | 2.67±0.7 | 2.71±0.7 | 0.2 | 0.3 |
| TyG index | 8.24±0.41 | 8.31±0.42 | <0.001 | 0.01 |
| RC (mmol/l) | 0.47±0.20 | 0.49±0.22 | 0.02 | 0.2 |
| VD (nmol/l) | 61.3±7.7 | 28.6299 9.61366 | <0.001 | <0.001 |

Note: Data are presented as the means ± SDs, and the p value is significant at the 0.05 level. BMI, body mass index; SBP, systolic blood pressure; DBP, diastolic blood pressure; TG, triglycerides; TC, total cholesterol; HDL-C, high-density lipoprotein cholesterol; LDL-C, low-density lipoprotein cholesterol; FG, fasting glucose; TyG index, triglyceride-glucose index; RC, remnant cholesterol; VD, vitamin D.

S. Table 6. Correlation matrices between TyG, RC, VD, and cardiometabolic risk factors

|  | | TgG | RC | VD | BMI | SBP | DBP | TG | TC | HDL-C | LDL_C |  |
| --- | --- | --- | --- | --- | --- | --- | --- | --- | --- | --- | --- | --- |
| TgG | Pearson Correlation | 1 | .878^**^ | -.050^**^ | .219^**^ | .071^**^ | .012 | .941^**^ | .163^**^ | -.207^**^ | .000 |  |
|  | Sig. (2-tailed) |  | .000 | .001 | .000 | .000 | .410 | .000 | .000 | .000 | .975 |  |
|  | N | 4865 | 4865 | 4730 | 4865 | 4865 | 4865 | 4865 | 4865 | 4865 | 4865 |  |
| RC | Pearson Correlation | .878^**^ | 1 | -.032^*^ | .217^**^ | .042^**^ | -.012 | .922^**^ | .228^**^ | -.225^**^ | .041^**^ |  |
|  | Sig. (2-tailed) | .000 |  | .028 | .000 | .003 | .384 | .000 | .000 | .000 | .005 |  |
|  | N | 4865 | 4865 | 4730 | 4865 | 4865 | 4865 | 4865 | 4865 | 4865 | 4865 |  |
| VD | Pearson Correlation | -.050^**^ | -.032^*^ | 1 | -.079^**^ | -.059^**^ | -.078^**^ | -.045^**^ | -.002 | .040^**^ | -.009 |  |
|  | Sig. (2-tailed) | .001 | .028 |  | .000 | .000 | .000 | .002 | .878 | .006 | .544 |  |
|  | N | 4730 | 4730 | 4730 | 4730 | 4730 | 4730 | 4730 | 4730 | 4730 | 4730 |  |
| BMI | Pearson Correlation | .219^**^ | .217^**^ | -.079^**^ | 1 | .306^**^ | .154^**^ | .215^**^ | .075^**^ | -.189^**^ | .087^**^ |  |
|  | Sig. (2-tailed) | .000 | .000 | .000 |  | .000 | .000 | .000 | .000 | .000 | .000 |  |
|  | N | 4865 | 4865 | 4730 | 4865 | 4865 | 4865 | 4865 | 4865 | 4865 | 4865 |  |
| SBP | Pearson Correlation | .071^**^ | .042^**^ | -.059^**^ | .306^**^ | 1 | .469^**^ | .042^**^ | -.001 | -.146^**^ | .039^**^ |  |
|  | Sig. (2-tailed) | .000 | .003 | .000 | .000 |  | .000 | .004 | .942 | .000 | .007 |  |
|  | N | 4865 | 4865 | 4730 | 4865 | 4865 | 4865 | 4865 | 4865 | 4865 | 4865 |  |
| DBP | Pearson Correlation | .012 | -.012 | -.078^**^ | .154^**^ | .469^**^ | 1 | .000 | .055^**^ | -.056^**^ | .085^**^ |  |
|  | Sig. (2-tailed) | .410 | .384 | .000 | .000 | .000 |  | .996 | .000 | .000 | .000 |  |
|  | N | 4865 | 4865 | 4730 | 4865 | 4865 | 4865 | 4865 | 4865 | 4865 | 4865 |  |
| TG | Pearson Correlation | .941^**^ | .922^**^ | -.045^**^ | .215^**^ | .042^**^ | .000 | 1 | .128^**^ | -.223^**^ | -.044^**^ |  |
|  | Sig. (2-tailed) | .000 | .000 | .002 | .000 | .004 | .996 |  | .000 | .000 | .002 |  |
|  | N | 4865 | 4865 | 4730 | 4865 | 4865 | 4865 | 4865 | 4865 | 4865 | 4865 |  |
| TC | Pearson Correlation | .163^**^ | .228^**^ | -.002 | .075^**^ | -.001 | .055^**^ | .128^**^ | 1 | .294^**^ | .930^**^ |  |
|  | Sig. (2-tailed) | .000 | .000 | .878 | .000 | .942 | .000 | .000 |  | .000 | .000 |  |
|  | N | 4865 | 4865 | 4730 | 4865 | 4865 | 4865 | 4865 | 4865 | 4865 | 4865 |  |
| HDL-C | Pearson Correlation | -.207^**^ | -.225^**^ | .040^**^ | -.189^**^ | -.146^**^ | -.056^**^ | -.223^**^ | .294^**^ | 1 | .037^**^ |  |
|  | Sig. (2-tailed) | .000 | .000 | .006 | .000 | .000 | .000 | .000 | .000 |  | .010 |  |
|  | N | 4865 | 4865 | 4730 | 4865 | 4865 | 4865 | 4865 | 4865 | 4865 | 4865 |  |
| LDL-C | Pearson Correlation | .000 | .041^**^ | -.009 | .087^**^ | .039^**^ | .085^**^ | -.044^**^ | .930^**^ | .037^**^ | 1 |  |
|  | Sig. (2-tailed) | .975 | .005 | .544 | .000 | .007 | .000 | .002 | .000 | .010 |  |  |
|  | N | 4865 | 4865 | 4730 | 4865 | 4865 | 4865 | 4865 | 4865 | 4865 | 4865 |  |
| **. Correlation is significant at the 0.01 level (2-tailed). | | | | | | | | | | | | |
| *. Correlation is significant at the 0.05 level (2-tailed). | | | | | | | | | | | | |
